# Associations between endometrial swab bacteriology and cytology findings and live foal rates in Thoroughbred broodmares in the United Kingdom

**DOI:** 10.1111/evj.70086

**Published:** 2025-09-01

**Authors:** Billy Fehin, Camilla J. Scott, Juan Carlos Arango‐Sabogal, Amanda M. de Mestre, Rebecca Mouncey

**Affiliations:** ^1^ Rossdales Veterinary Surgeons Beaufort Cottage Stables Suffolk UK; ^2^ Département de pathologie et microbiologie Faculté de médecine vétérinaire, Université de Montréal Saint‐Hyacinthe Québec Canada; ^3^ Department of Pathobiology and Population Sciences The Royal Veterinary College, University of London Hertfordshire UK; ^4^ Baker Institute for Animal Health, College of Veterinary Medicine Cornell University Ithaca New York USA

**Keywords:** culture, cytology, endometrial, fertility, horse, Thoroughbred

## Abstract

**Background:**

To date, relationships between pre‐covering endometrial swab cytology and bacteriology and fertility outcomes in Thoroughbred broodmares in the United Kingdom are unknown and could aid clinical decision making.

**Objectives:**

To investigate associations between cytology and bacteriology findings from the last endometrial swab taken in the breeding season (15 February to 15 July) and live‐foal rates (predicted mean probability of producing a live foal) in UK Thoroughbred broodmares.

**Study Design:**

Retrospective cohort study.

**Methods:**

Endometrial cytology and bacteriology findings were extracted from laboratory records for all last‐swabs submitted between 2014 and 2020. Mares' status, age and foaling outcome were collected from publicly available data sources. Live‐foal rates were estimated for reported categories of cytology and bacteriology findings using a multivariable logistic regression model with mare and farm fitted as random effects, while adjusting for mares' age, status, number of previous swabs submitted in that season and any interactions. Pairwise comparisons with Bonferroni correction evaluated between‐category live‐foal rate differences.

**Results:**

Data were available from 7691 last swabs from 3579 mares on 196 farms. In contrast to other categories of isolate, mares with a profuse growth of *Escherichia coli* had significantly lower (*p* = 0.005) live‐foal rates (59.1%; 95% confidence interval (CI) 43.7–74.5) compared to those with no growth (80.9%; 95% CI 79.2–82.6). There was interaction between mares' age and cytology. In mares >12 years, significant reductions in live‐foal rates (*p* < 0.05 in pairwise comparisons) were observed between mares with >30% polymorphonuclear: endometrial cells/high power field at cytological examination and mares with ≤0.5% PMN, a finding absent in mares ≤12 years.

**Main Limitations:**

The use of unguarded swabs and absence of clinical information.

**Conclusions:**

Results highlight complexities to consider when interpreting endometrial swab cytology findings and a subset of mares with a profuse growth of *E. coli* in which knowledge gaps exist around the aetiologies underlying their poorer fertility outcomes.

## Résumé

**Contexte:**

À ce jour, la relation entre les cytologies et bactériologies sur écouvillons endométriaux pré‐saillie et les taux de fertilité chez les juments poulinières au Royaume‐Uni demeurent inconnus et pourrait contribuer au raisonnement clinique.

**Objectifs:**

Investiguer les associations entre les résultats cytologique et bactériologique provenant du dernier écouvillon prélevé en saison de reproduction (15 Février au 15 Juillet) et les taux de naissance de poulains vivants (probabilité moyenne prédite de produire un poulain vivant) chez les juments poulinières au Royaume‐Uni.

**Modèle d’étude:**

Étude de cohorte rétrospective.

**Méthodes:**

Les résultats de cytologie et bactériologie endométriales ont été retrouvés parmi les dossiers de laboratoire pour tous les derniers écouvillons soumis entre 2014 et 2020. Le statut des juments, leur âge et leur succès au poulinage out été recueillis à partir de données disponibles au public. Les taux de poulains vivants ont été estimés pour les catégories de résultats cytologiques et bactériologiques en utilisant un modèle de régression linéaire avec jument et ferme identifiés comme effets aléatoires et en ajustant pour le statut des juments, leur âge, le nombre d’écouvillons soumis précédemment dans la saison de reproduction et toutes interactions. Des comparaisons en paires avec correction de Bonferroni ont été utilisées pour évaluer les différences entre les catégories pour les taux de poulains vivants.

**Résultats:**

Les données de 7691 écouvillons provenant de 3579 juments sur 196 fermes ont été retrouvées. Au contraire des autres catégories de souches, les juments avec croissance profuse d’*Escherichia coli* avait un taux de poulains vivants significativement inférieur (*P* = 0.005, 59.1%; 95% confidence interval (CI) 43.7–74.5) comparativement aux juments avec aucune croissance (80.9%; 95% CI 79.2–82.6). Une interaction entre l’âge des juments et la cytologie a été identifiée. Pour les juments de plus de 12 ans, des réductions significatives du taux de poulains vivants ont été observées entre les juments ayant plus de 30% de cellules endométriales polymorphonucléaires/haut champs à l’examen cytologique et celles avec ≤0.5% PMN, un résultat absent chez les juments de ≤12 ans.

**Limites Principales:**

Utilisation d’écouvillon sans gaine protectrice et absence d’information clinique.

**Conclusions:**

Les résultats suggèrent certaines complexités lors d’interprétation des résultats de cytologie pour les écouvillons endométriaux et une sous‐population de juments avec croissance profuse d’*Escherichia coli* pour qui des incompréhensions persistent quant aux étiologies potentielles pour leur fertilité réduite.

## 1 INTRODUCTION

In the United Kingdom the Horseracing Betting Levy Board (HBLB) Codes of Practice^1^ require that Thoroughbred broodmares are certified free of the venereal pathogens: *Taylorella equigenitalis* (CEMO), *Klebsiella pneumoniae* (capsule types 1, 2 and 5) and *Pseudomonas aeruginosa*, via an endometrial swab taken in oestrus prior to every cover. As a result, large numbers of pre‐breeding samples from clinically normal mares are sent for aerobic and microaerophilic bacteriological culture, where cytological evaluation and the culture of any other microbial isolates in addition to the above venereal pathogens are commonly undertaken. However, currently available commercial testing does not differentiate whether any microbes identified are pathogens, contaminants or part of the normal commensal uterine microbiome.^2^, ^3^ With the absence of a ‘gold standard’ test for bacterial endometritis, there is significant potential for misclassification errors.^4^

Bacterial endometritis typically occurs in mares susceptible to persistent breeding induced endometritis (PBIE) that have an inadequate uterine immune response and impaired ability to resolve physiological inflammation and uterine clear fluid following mating.^5^, ^6^ As a result, pathogenic and/or opportunistic microorganisms entering the uterus at breeding,^5^, ^6^ such as *Streptococcus equi* subspecies *zooepidemicus* and *Escherichia coli* can cause PBIE to progress to bacterial endometritis, which is considered to be an important cause of infertility.^5^ As such, clinicians are often faced with difficult decisions around which mares should present for cover or receive antimicrobials. With the pressure of producing a live foal every year and a short breeding season, historically empirical post cover treatment with intrauterine antimicrobials was commonplace.^7^, ^8^ In an age of antimicrobial stewardship and increasing evidence demonstrating potential detrimental effects of antimicrobial usage, there is a need for better understanding of the significance of findings from endometrial swabs.^9^, ^10^

Understanding the relationship between endometrial swab findings and fertility outcomes may inform decision making. Studies from the United Kingdom are lacking, but in the United States, Riddle et al.^11^ reported that mares with positive cytology (>20% polymorphonuclear cells/high power field) or culture (any microorganism recovered) had lower 28‐day pregnancy rates than mares without these findings. Similarly, Davies Morell et al.^12^ reported a reduction in live‐foal rates with either positive cytology alone (≥1% PMN), positive bacterial culture alone (monoculture of any isolate) or a combination of both positive cytology and bacteriology, although the significance of these relationships varied depending on whether all swabs, the first swab, or the last swab taken in the breeding season were evaluated.^12^ Isolate prevalence is recognised to vary not only between populations and geographical locations, but also between mares of different ages and status.^13^ Factors which were unaccounted for by these studies' univariable analysis methods are importantly also known to influence fertility outcomes.^14^, ^15^

The aim of the present study was, therefore, to use multivariable modelling methods to investigate associations between endometrial swab findings and live‐foal rates in UK Thoroughbred broodmares. Specific objectives were to investigate associations between endometrial swab bacteriology and cytology findings from the last endometrial swab submitted in the northern hemisphere breeding season (15 February to 15 July) and the probability of producing a live foal (live‐foal rate), while accounting for mares' age, status at the start of the breeding season and number of previous endometrial swab samples submitted in that season. We hypothesised that mares with a positive cytology and/or a positive bacterial culture would have lower live‐foal rates.

## 2 MATERIALS AND METHODS

This study forms part of a larger body of work that has described the prevalence of microbial isolates,^13^ described antimicrobial resistance patterns in a subset of these isolates^16^ and evaluated current diagnostic strategies for endometritis.^4^

### 2.1 Sample size calculation

Sample size calculations were performed in Stata (release 16; Stata Corp. LP). Assuming a live‐foal rate of 80% in the unexposed group,^8^ a total sample size of between 658 and 3698 swabs can detect odds ratios (OR) between 0.6 and 0.8 with 80% power (and 0.05 alpha error).

### 2.2 Data collection

Details of all endometrial swab samples from Thoroughbred mares submitted to Rossdales Laboratories, Newmarket, between 2014 and 2020 were collected from the laboratory's database. Details of the mares' date of birth, status at the start of the breeding season (categorised as either (i) Foaling, (ii) Maiden, (iii) Rested; not covered, (iv) Barren; covered but not pregnant or (v) Aborted; pregnancy loss at any stage of gestation) and foaling outcome for each season (categorised as either (i) Live foal, (ii) Dead foal, (iii) Mare died, (iv) Rested, (v) Barren or (vi) Aborted) were collected, where available, from publicly available data sources.^17^, ^18^ All data were entered into Excel.

### 2.3 Sample collection and laboratory analysis

Details of sample collection and laboratory analyses are described in detail elsewhere.^4^, ^13^, ^16^

Briefly, endometrial swabs were collected during oestrus. Following aseptic preparation of the perineum, a single‐use speculum was inserted into the vagina, through which two unguarded swabs were inserted sequentially into the uterus via the cervix. The first was placed into charcoal medium for microbial culture, and the second into a plain sheath for cytology and transported unrefrigerated to the laboratory and either plated (charcoal medium) or smeared (plain swab) within 12 h of collection.

Cultures were plated onto Blood, MacConkey and Chocolate Blood agar for aerobic and anaerobic culture, respectively. Plates were kept at 37°C and checked after 12–24 h and again at 48 h. Colonies were identified using the Analytical Profile Index (bioMérieux, France) and reported when at least one colony was identified. Growth was classified as either (i) a few colonies: less than 10 colonies, (ii) moderate: gaps can be identified between colonies, or (iii) profuse: no gaps can be identified between colonies.

For cytology, smears were stained with Pollack's Rapid Trichrome stain on the Leica Autostainer XL. The whole slide was scanned, and results averaged and graded based on the percentage of polymorphonuclear neutrophils (PMN) cells: endometrial cells per high power field^19^ as: 0 (No PMN); +/− (≤0.5% PMN); 1+ (>0.5%–5% PMN); 2+ (>5%–30% PMN); and 3+ (>30% PMN).

### 2.4 Data processing

All data were imported into Stata (Release 16, StataCorp LP). Swabs for which there were no foaling outcomes at the end of the respective season and those taken outside of the northern hemisphere breeding season (15 February to15 July) were excluded from analyses. Swab data were then sorted by mare, year (season) and date of sampling. The study sample was generated by using the sampling date to identify the last swab taken in each breeding season for each mare. A variable was created for each mare in each breeding season to indicate the total number of endometrial swabs submitted to the laboratory in that season prior to the last swab of the same season. The mares' age at the start of the breeding season was calculated by subtracting the year of sampling from her year of birth (using date of birth data and date of sampling data). Bacteriology findings were categorised by isolate and amount of growth as described in the pathologist's report. Foaling outcomes were further categorised to generate a binary outcome variable indicating whether or not the mare produced a live foal at the end of the breeding season (i.e., the outcomes of Dead‐foal, Barren and Aborted were categorised as no live foal). Mares that were rested or died during the breeding season were excluded from analyses with their respective outcome variables left blank in the respective season, as they had no opportunity to produce a live foal in that season. The study sample therefore included observations that corresponded to mares' last endometrial swab in each season, which included the bacteriology and cytology findings, her status, age, and farm on which she resided at the start of that season, the number of previous endometrial swabs submitted to the laboratory in that season, and her binary foaling outcome for that season.

### 2.5 Data analysis

All analyses were performed in Stata (Release 16, StataCorp LP). A description of the study sample was performed, including the number of samples, mares, and farms by year of sampling. Histograms were plotted of continuous variables (mares' age and number of previous swabs) and visually inspected for normality. Normally distributed data were described by mean, standard deviation (SD) and range; non‐normally distributed data were described by median, interquartile range (IQR) and range. Proportions and 95% confidence intervals (CI) were calculated for categorical data.

**FIGURE 1:**
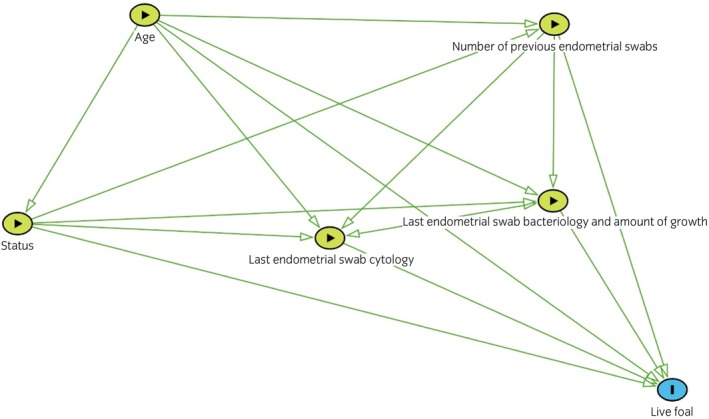
A directed acyclic graph (DAG) to identify the minimal sufficient adjustment set of variables to investigate associations between cytology and bacteriology findings from the last endometrial swab taken in the breeding season and live‐foal rates (predicted mean probability of producing a live foal) in UK Thoroughbred broodmares.

Mixed effects multivariable logistic regression modelling was used to investigate associations between endometrial swab cytology and bacteriology findings and the odds of producing a live foal. Due to the clustered nature of the data,^20^ farm and mare were fitted a priori as random effects to all models. The shape of the association between continuous predictor variables and the outcome of interest was explored using likelihood ratio test (LRT) for departure from linear trend. Variables with LRT *p* < 0.05 were modelled as categorical (quartiles). A directed acyclic graph (DAG)^21^ was constructed a priori to estimate the total effects of the fixed exposures of interest (last endometrial swab bacteriology and cytology alongside mares' age, status and number of previous endometrial swabs taken in that breeding season) on the outcome to inform analyses (Figure 1). The DAG highlighted that there was potential for both interaction and confounding. Univariable analysis was undertaken of all predictor variables to obtain unadjusted odds ratio (OR) estimates. To evaluate confounding, the final model was constructed by stepwise inclusion of all fixed predictor variables. There was deemed to be evidence of confounding if OR estimates changed by more than 20% when predictor variables were added to the model. Pairwise interactions were tested between all predictor variables. Evidence of interaction was considered present if the LRT comparing a model with and without the interaction term resulted in *p* < 0.05. Significant interaction terms were retained in the final model as appropriate.

To estimate the effects of endometrial swab findings on live foal rates, allow inference to the total population from which data were drawn, and further understand relationships in the final model,^22^ predicted margins^23^ were calculated using Stata *margins* suite of post‐estimation commands.^24^ This procedure allowed us to estimate the predicted mean probability of producing a live foal (live‐foal rate) for each level of each predictor variable, while accounting for the effect of all other variables and interaction terms in the model. Where appropriate, pairwise comparisons of predicted probabilities were performed to test for differences between categories within predictor variables; a Bonferroni correction was applied to correct for multiple comparisons. Statistical significance was set at *p* < 0.05.

The normality and homoscedasticity of the residuals at the highest level (farm) were confirmed graphically using Q–Q plots and plots of residuals against the predicted values; the fit of the final model over the range of data was assessed graphically by plotting the sum of the observed against predicted values in each decile of predicted probability.

## 3 RESULTS

A flowchart providing details of the initial database, data exclusions, study sample and final model is provided in Figure S1. The initial database consisted of 18,996 endometrial swabs collected from 6050 mares on 290 farms over 7 breeding seasons (2014–2020). The source population consisted of 7691 last swabs from 3579 mares on 196 farms submitted over the 7 breeding seasons. The distribution of last swabs, mares and farms by year of sampling is provided in Table S1.

### 3.1 Description of the source population

Within the source population, the median number of endometrial swabs submitted prior to the last swab, per mare per season, was 0; IQR 0–1, range 1–10. Mare age at the start of the breeding season data were available for 6845 of the 7691 last swabs; the median age was 8 years, IQR 6–12, range 2–24. Status at the start of the breeding season data were available for 6988 of the 7691 last swabs; the distribution of mares' status at the start of the breeding season is displayed in Table 1.

**TABLE 1:** The distribution of mares' status at the start of the breeding season for the 6988 endometrial swab samples for which status data were available, from 7691 last endometrial swab samples submitted during the breeding season (15 February to 15 July) from 3579 Thoroughbred mares on 196 farms over 7 breeding seasons, to a laboratory in Newmarket, UK between 2014 and 2020.

Status	*n*	%	95% confidence interval
Foaling	4877	69.8	68.7–70.9
Barren	1102	15.8	14.9–16.6
Maiden	898	12.8	12.1–13.7
Rested	105	1.5	1.3–1.9
Aborted	6	0.1	0.04–0.2
Total	6988		

*Note*: Barren = covered not pregnant; Rested = not covered; Aborted = pregnancy lost at any time during gestation.

Of the 7691 last swabs, 1808 (23.5%; 95% CI 22.6–24.5) were reported to have had bacterial growth. Beta haemolytic *streptococcus* (BHS) in monoculture (10% of last swabs; 95% CI 10.2–11.6) and *E. coli* in monoculture (5.5%; 95% CI 5.1–6.1) were the most frequently reported isolates. The distribution of the most frequently reported isolates and amount of growth, with sufficient prevalence to be used for modelling purposes, is reported in Table 2; a description of all microorganism species cultured from the source population is reported elsewhere.^13^

**TABLE 2:** The distribution of the most frequently reported bacterial isolates and amount of growth from 7691 last endometrial swab samples collected during the breeding season (15 February to 15 July) from 3579 Thoroughbred mares on 196 farms, submitted to a laboratory in Newmarket, UK between 2014 and 2020.

Bacteriology	*n*	%	95% confidence interval
No growth	5883	82.3	81.4–83.2
BHS mono, few colonies	629	8.8	8.2–9.5
BHS mono, moderate	115	1.6	1.3–1.9
BHS mono, profuse	91	1.3	1.0–1.6
EC mono, few colonies	302	4.2	3.8–4.7
EC mono, moderate	66	0.9	0.7–1.2
EC mono, profuse	59	0.8	0.6–1.1
Total	7145		

Abbreviations: BHS, beta haemolytic *streptococcus*; EC, *Escherichia coli*; moderate, moderate growth reported by the pathologist; mono, monoculture; profuse, profuse growth reported by the pathologist.

Cytology findings were available for 7630 of the 7691 last swabs. The distribution of endometrial cytology findings is presented in Table 3. The distribution of endometrial cytology and bacteriology findings for the most frequently reported categories of bacterial isolate and growth is presented in Table S2.

**TABLE 3:** The distribution of reported endometrial swab cytology findings for the 7630 swabs for which cytology findings were available, from 7691 last endometrial swab samples collected during the breeding season (15 February to 15 July) submitted from 3579 Thoroughbred mares on 196 farms, over 7 breeding seasons to a laboratory in Newmarket, UK between 2014 and 2020.

Cytology	*n*	%	95% confidence interval
0	6521	85.5	84.7–86.2
+/−	713	9.3	8.7–10.0
1+	207	2.7	2.4–3.1
2+	102	1.3	1.3–1.6
3+	87	1.1	0.9–1.4
Total	7630		

*Note*: 0 = no polymorphonuclear neutrophils (PMN) cells; +/− = ≤0.5% PMN; 1+ = >0.5%–5% PMN; 2+ = >5%–30% PMN; 3+ = >30% PMN:endometrial cells per high power field.

**FIGURE 2:**
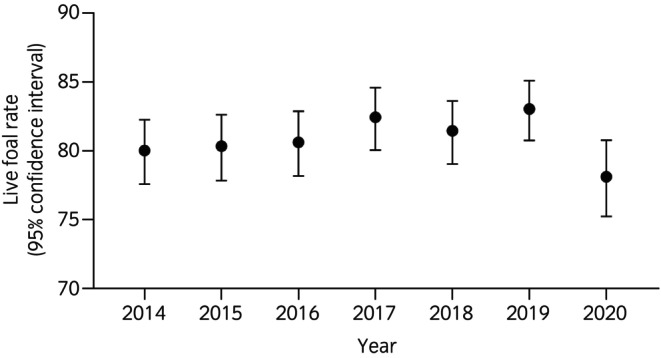
The live foal rate by year (proportion of mares reported to have produced a live foal) for 3597 Thoroughbred mares on 196 stud farms that submitted 7691 endometrial swab samples over 7 seasons to a laboratory in Newmarket, UK, between 2014 and 2020.

The distribution of reported outcomes at the end of the breeding season, stratified by year, is provided in Table S3. The overall live‐foal rate for the study period was 81.0% (95% CI 80.0–81.8). The live‐foal rate by year is provided in Figure 2. There were no significant differences in live‐foal rates between years.

### 3.2 Associations with live‐foal rates

**FIGURE 3:**
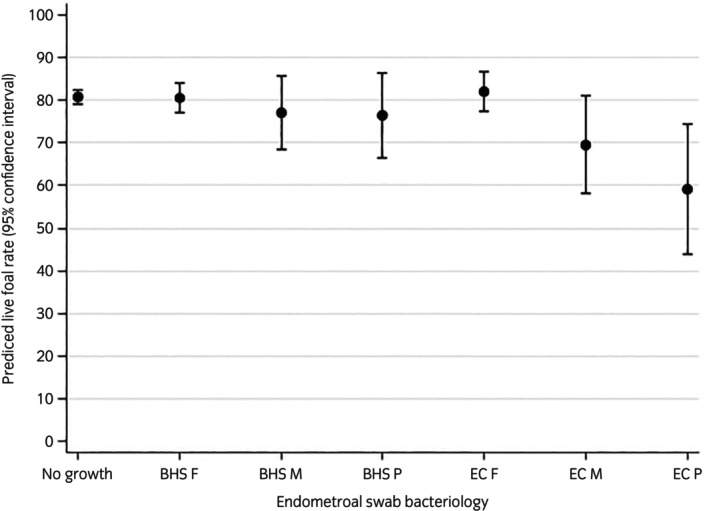
The predicted live foal rate (marginal predicted probability of producing a live foal) and 95% confidence intervals by category of mares' last endometrial swabs of the breeding seasons bacteriology findings. Estimates were obtained using a mixed effects multivariable logistic regression model on data from 5695 endometrial swabs from 2534 Thoroughbred mares on 145 stud farms submitted during the northern hemisphere breeding season (15 February and 15 July) to a laboratory in Newmarket, UK, between 2014 and 2020. BHS, beta haemolytic *streptococcus* in monoculture; EC, *Escherichia coli* in monoculture; F, few colonies; M, moderate growth; P, profuse growth.

**FIGURE 4:**
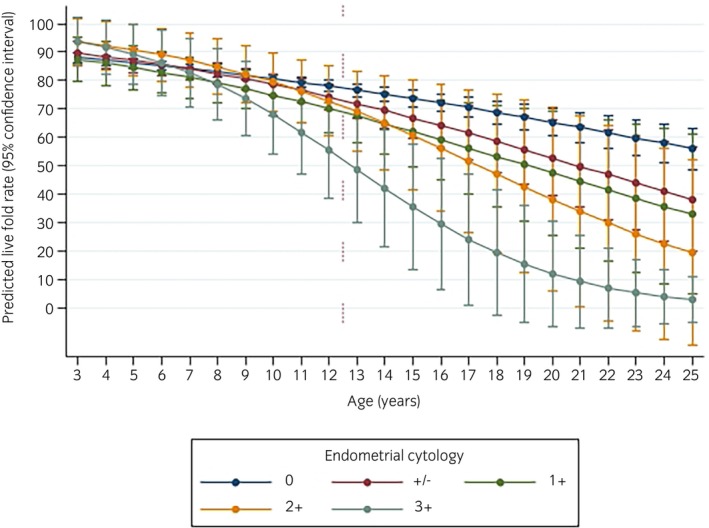
The predicted live foal rate (marginal predicted probability of producing a live foal) and 95% confidence intervals by mares' age and last endometrial swab of the breeding seasons' cytology finding. Estimates were obtained using a mixed effects multivariable logistic regression model on data from 5695 endometrial swabs, from 2534 Thoroughbred mares on 145 stud farms submitted during the northern hemisphere breeding season (15 February and 15 July) to a laboratory in Newmarket, UK, between 2014 and 2020. 0 = no polymorphonuclear neutrophils (PMN) cells; +/− = ≤0.5% PMN; 1+ = 0.5%–5% PMN; 2+ = >5%–30% PMN; 3+ = >30% PMN:endometrial cells per high power field.

Relationships in the final model were complex, with significant interaction and evidence of confounding. Mares' age, status and previous number of endometrial swabs submitted in that season were identified as potential confounders of the relationships between mares' endometrial cytology and bacteriology findings and the live foal rate. Predicted live‐foal rates with 95% CI for all predictor variables and interaction terms from the final model are described and presented below in Figures 3 and 4. The results of univariable analyses for all predictor variables are presented in Table S4, and the final multivariable model is presented in Table 4.

**TABLE 4:** Results of multivariable mixed effects logistic regression model to investigate associations between endometrial swab findings and live foal rates in 3579 Thoroughbred mares on 196 stud farms that submitted 7691 endometrial swab samples during the northern hemisphere breeding season (15 February and 15 July) to a laboratory in Newmarket, UK, between 2014 and 2020.

Predictor	Category	OR	95% CI	Wald *P*
Number of previous endometrial swabs	0	*Ref*	–	–
	1	0.87	0.73–1.04	0.14
	2	0.40	0.32–0.50	<0.001
	3–10	0.17	0.13–0.22	<0.001
Endometrial swab cytology	0	*Ref*	–	–
	+/−	1.34	0.0–2.58	0.37
	1+	1.08	0.40–3.06	0.89
	2+	2.96	0.47–18.6	0.25
	3+	3.97	0.54–29.32	0.18
Endometrial swab cytology#Age	0	*Ref*	–	–
	+/−	0.96	0.90–1.01	0.13
	1+	0.95	0.87–1.04	0.34
	2+	0.89	0.76–1.03	0.13
	3+	0.81	0.67–0.97	0.02
Age		0.91	0.90–0.93	<0.001
Status	Foaling	*Ref*	–	–
	Barren	1.65	1.31–2.08	<0.001
	Rested	2.51	1.29–4.88	0.007
	Maiden	1.10	0.83–1.46	0.49
	Aborted	2.40	0.22–26.1	0.47
Endometrial swab bacteriology	No growth	*Ref*	–	–
	BHS mono few	0.98	0.75–1.26	0.87
	BHS mono mod	0.77	0.43–1.36	0.37
	BHS mono prof	0.74	0.39–1.41	0.36
	EC mono few	1.08	0.76–1.57	0.64
	EC mono mod	0.49	0.26–0.92	0.03
	EC mono prof	0.29	0.14–0.60	0.001
Variance component	Farm	0.06	0.02–0.19	
	Mare	0.23	0.08–0.67	

*Note*: Age is modelled as a continuous variable, # indicates interaction term.

Abbreviations: BHS, beta haemolytic *Streptococcus*; CI, confidence interval; EC, *Escherichia coli*; few, few colonies; mod, moderate growth; mono, monoculture; OR, odds ratio; prof, profuse growth; *Ref*, reference category.

#### 3.2.1 Endometrial bacteriology

A graph of the predicted live‐foal rate for the most frequently reported categories of endometrial bacteriology finding is presented below in Figure 3. Compared to mares with an endometrial swab with no growth reported (predicted live‐foal rate 80.9%; 95% CI 79.2–82.6), mares with a profuse growth of *E. coli* in monoculture on their last swab in the breeding season had significantly lower (*p* = 0.005) predicted live‐foal rates (59.1%; 95% CI 43.7–74.5). Differences in predicted live‐foal rates between other categories of the most frequently reported bacteriology finding, including those with a profuse growth of Beta haemolytic *streptococcus*, were not significantly different.

#### 3.2.2 Endometrial cytology

There was significant interaction (*p* = 0.02) between mares' age and cytology findings, meaning that the effect of mares' cytology findings on the odds of producing a live foal varied depending on the age of the mare. The interaction between mares' age and endometrial cytology findings from the last swab submitted in the breeding season is presented graphically in Figure 4. In mares >12 years of age, significant reductions in live‐foal rates (*p* < 0.05; global alpha in pairwise comparisons) were observed between mares with >30% PMN and mares with ≤0.5% PMN. Differences in predicted live‐foal rates between all other categories of endometrial cytology in mares >12 years were not significantly different. In mares ≤12 years, live‐foal rates were not significantly different between categories of PMN.

#### 3.2.3 Mares' age, status and previous endometrial swabs

Live‐foal rate was observed to decrease with increasing mare's age, with an estimated reduction of −0.01% per year increase in age (95% CI −0.02 to −0.01; *p* < 0.001). Rested and Barren (89.4%; 95% CI 83.7–94.9 and 85.3%; 95% CI 82.9–87.7, respectively) mares were observed to have significantly higher (*p* < 0.05 in pairwise comparisons) live‐foal rates compared to Foaling (79.0%; 95% CI 77.1–80.9) mares. Mares that had two (71.0%; 95% CI 67.2–74.8) or 3–12 (53.5%; 95% CI 48.0–59.0) previous endometrial swabs submitted during the breeding season were observed to have significantly lower (*p* < 0.05 in pairwise comparisons) predicted live‐foal rates compared to those having either none (85.0%; 95% CI 83.2–86.7) or just one (83.3%; 95% CI 81.1–85.5) previous endometrial swab submitted.

The normality and homoscedasticity of the residuals are demonstrated graphically in Figure S2 in a Q–Q plot and plot of residuals against the predicted values, and acceptable fit of the final model over the range of data is demonstrated in Figure S3 in a plot of the sum of the observed against predicted values in each decile of predicted probability.

## 4 DISCUSSION

This is the first study to use multivariable modelling methods to investigate associations between endometrial swab findings and live‐foal rates in Thoroughbred broodmares, identifying novel and important relationships. Endometrial bacteriology and cytology findings from the last endometrial swab of the breeding season were associated with the odds of producing a live foal; however, relationships were complex with confounding and interaction detected, demonstrating the importance of considering the combined effects of these factors both when interpreting endometrial swab findings and evaluating fertility outcomes in this population.

Utilisation of predicted margins,^24^ to estimate the effects of endometrial swab findings on live‐foal rates, while accounting for the effects of mares' age, status and previous number of endometrial swab samples submitted in the season, provides novel estimates that can help inform clinical decision making.

In contrast to all other categories of endometrial bacteriology findings (including those with a profuse growth of Beta haemolytic *streptococcus*) mares that had a profuse growth of *E. coli* in monoculture from their last swab submitted in the breeding season had significantly lower predicted live‐foal rates compared to mares with no growth. Only two previous studies, both from the United States, have evaluated the effect of endometrial bacteriology and cytology findings on fertility outcomes in Thoroughbred broodmares.^11^, ^12^ Both of these studies have important limitations compared to the present work. First, due to their univariable analysis methods, they fail to account for the effects of other factors such as mares' age, status and cytology findings, which are recognised to influence both fertility outcomes and endometrial swab findings in these poulations.^14^, ^15^ Second, because they evaluated the effects of all species of bacteria simultaneously, their analyses do not account for any differences in host‐pathogen dynamics and response to treatment between bacterial species, which may also influence fertility outcomes. Therefore, estimates from the present study provide additional insight and nuance to previously described relationships between endometrial swab bacteriology findings and fertility outcomes.

Davies Morel et al.,^12^ hypothesised, as a reason for their observed poorer fertility outcomes with gram‐negative compared to gram‐positive bacteria, that biofilm formation by gram‐negative bacteria may have provided protection against host immune responses and limited antimicrobial penetration,^25^ meaning that gram‐negative bacteria were not effectively eliminated despite antimicrobial treatment. The virulence of *E. coli* isolates depends on the coordinated expression of several virulence factors including the ability to adhere to the host endometrium, form a biofilm and infer antimicrobial resistance.^26^ *Escherichia coli* isolates collected from the equine reproductive tracts of mares have previously shown the ability to form and exist in a biofilm state; however, the clinical relevance of this has not yet been fully determined.^27^ Clinical information was not available for mares in the present study, meaning that clinical findings or details of any treatments are unknown, which is an important limitation of this work. The identification of *E. coli* on an endometrial swab may be considered a contaminant by some clinicians, particularly due to the use of unguarded swabs; however, a profuse growth in monoculture would most likely be thought to be clinically significant. Despite potential differences among veterinary clinicians in their implementation of uterine treatments, any treatments that may have been received did not improve the live‐foal rate in this *E. coli* group compared to the rest of the mares in the study, highlighting important fertility differences in this subset of mares. Further investigations are therefore required to identify the aetiology underlying the poorer fertility outcomes and understand the role that different strains of *E. coli* may play; why they may not respond to current treatments, and what factors potentially allow for the persistence of this bacteria.

Our previous work demonstrated that the majority of variance in *E. coli* prevalence in this population is observed at the mare‐level,^13^ suggesting that the presence of this isolate is mainly due to factors intrinsic to the individual mare, which are less likely to change over time between samples. Given that the general consensus now is that culture‐based technologies detect less than 10% of the resident microbial community in a sample,^28^ an additional hypothesis could be that the *E. coli* isolated in this group may represent a potential biomarker of disruption to the normal balance of the uterine microbiota, leading to dysbiosis and facilitation of *E. coli* proliferation, resulting in endometrial inflammation^29^ and poorer fertility outcomes. Antimicrobial resistance of *E. coli* isolates is another important factor that could account for the reduction in live‐foal rates in this subset of mares. In a study of antimicrobial resistance of a relatively small subset (*n* = 2091 isolates; 29% of all those reported) of present isolates,^16^ the majority (81.7%) of *E. coli* isolates were susceptible to gentamicin.^16^ However, it has been recently demonstrated that 50% of the strains of *E. coli* identified from the uterus of mares with fertility problems had the presence of selected virulence factors^27^ which could potentially affect their susceptibility to antimicrobial treatment in vivo. Present findings not only highlight important knowledge gaps around *E. coli* isolates, but also identify another important subset of mares with a profuse growth of *E. coli* and reduced fertility outcomes. In order to ensure antimicrobial stewardship, further work is urgently required to improve our understanding of the endometrial microbiome and allow us to unravel the complexities around whether such isolates are truly ‘resistant pathogens’ or in fact ‘bio‐markers’ of a syndrome of endometrial dysbiosis and dysfunction for which conventional antimicrobial therapy may be inappropriate.^30^

In contrast to those with a profuse growth of *E. coli*, mares with a profuse growth of Beta haemolytic *streptococcus* (BHS) on the last swab taken in the breeding season did not have significantly different live‐foal rates compared to mares with no growth. While endometritis caused by BHS may be difficult to diagnose and treat in a dormant state,^31^, ^32^ once detected, it is highly sensitive to penicillin^13^ and hence antimicrobial treatment is usually effective. Similarly, differences in predicted live‐foal rates between other categories of bacteriology findings, including a few or moderate growth of BHS and *E. coli*, were not significantly different from mares with no bacterial growth. In the absence of mares' clinical history, the significance of these few and moderate monocultures is unknown. However, given the limited specificity of a single endometrial swab to detect bacterial endometritis,^4^ coupled with the low prevalence of endometritis in a population of pre‐breeding samples, it is very possible that these represent false positive cultures from clinically normal mares, and as such, would not be expected to be associated with lower foaling rates. Something which clinicians must be mindful of, in terms of antimicrobial stewardship, when considering therapeutic rationale.

In the present study, an important and novel interaction was identified between mares' age and cytology findings. Endometrial inflammation (including marked; >30% PMN) appeared not to influence live‐foal outcomes in young (≤12 years old) mares. In contrast, marked inflammation (>30% PMN) in older mares (>12 years) was associated with significantly reduced live‐foal rates. These findings highlight the importance of evaluating the effects of age and cytology concurrently, both when interpreting endometrial swab findings and when evaluating poor fertility outcomes in this population. Previous studies^11^, ^12^ have also shown that mares with a positive cytology (either >21%^11^ or ≥1% PMN's^12^ of total cells) were associated with lower 28‐day pregnancy^11^ rates and foaling rates,^12^ compared to those of mares with no inflammatory cells on cytology. However, unlike the present work, these studies fail to account for any concurrent effects of mares' age on this association by only utilising univariable analysis methods.

It is well established that increases in the age of the mare have a negative effect on fertility outcomes.^33^, ^34^ Present findings, however, provide novel additional context to this by identifying an important subset of older mares with significant endometrial inflammation that were associated with poorer fertility outcomes (>12 years of age >30% PMN:endometrial cells/HPF). The work of Carnevale and Ginther,^35^ which compared uterine function and reproductive efficiency in groups of young (5–7 years) and old (≥15 years) mares, may help to elucidate potential mechanisms underlying the interaction observed between age and endometrial cytology in the present study. Carnevale and Ginther found that, compared to young mares, older mares had lower uterine contractility scores, a tendency for reduced uterine tone, more extensive intrauterine fluid collections on ultrasonography, and endometrial biopsies with more inflammatory cell infiltrations, more fibrotic changes, and less dense endometrial glands.^34^ Authors hypothesised that it was these important differences observed between young and older mares that led to the significantly lower (32% vs. 100%, respectively) Day 12 pregnancy rates and significantly higher (62% vs. 11%, respectively) embryo‐loss rates (Days 12–39) observed in the old mare group.^34^ For practitioners, present evidence therefore suggests that in older mares, where uterine clearance mechanisms are more likely to be suboptimal, the routine use of diagnostics to identify significant endometrial inflammation appears to be warranted. However, in younger mares, where endometrial cytology findings appear less likely to affect fertility outcomes, the routine use of such diagnostics may be harder to justify.

We also observed that rested and barren mares had significantly higher predicted live‐foal rates compared to foaling mares, suggesting that resting mares may be of benefit in terms of subsequent fertility outcomes. These findings are in contrast to previous studies, which reported that barren or rested status increased the likelihood of pregnancy loss^15^ and barren status reduced pregnancy rates.^36^ Clinical history was not available for mares in the present study, and it was therefore possible that barren and rested mares may have received treatment or barren mare investigations at the end of the previous season, which may have subsequently improved their live‐foal rates. Allowing time for any acute inflammation from PBIE to resolve rather than accumulate following repeated covers may have benefited rested mares, particularly in the subset of older mares with marked endometrial inflammation (>12 years, >30% PMN) that are likely to have poorer endometrial clearance capacity.^34^ Rested and barren mares may also have had more opportunities and a longer time frame in which to be covered, compared to foaling mares that may have only had one cover if they foaled later on in the season.

Mares that had two or more previous endometrial swabs submitted in the season were also observed to have significantly lower predicted live‐foal rates compared to those with only one or no previous swabs taken, most likely due to previous swabs reflecting the number of previous coverings (as HBLB codes of practice requiring a new endometrial swab before each cover). Again, the reproductive histories of these mares and the stallions used were unknown, but despite this, findings provide further weight of evidence that mares requiring multiple (three or more) covers may be likely to have reduced live‐foal rates and highlight a subset of mares in which resting could perhaps improve subsequent reproductive efficiency.

The main limitation of the present study was that information on the reproductive histories and treatment (if any) of the individual mares was not available. Without such clinical information, we are unable to determine if mares with positive cytologies and bacterial cultures had other signs of infectious or inflammatory endometritis. Treatment of these mares in response to the submitted endometrial swab results was also unknown, and while it is likely that mares with a profuse monoculture of bacteria were likely to have received antimicrobials, the drug choice, duration and route of treatment were unknown. Mares presenting with intrauterine fluid pre‐ and/or post‐cover would also have been likely in this population to have received adjunctive therapy such as uterine lavages, ecbolics and correction of any perineal defects. Again, the potential effects of these treatments are also unknown. Endometrial swabs were taken by multiple veterinary surgeons across multiple stud farms, and therefore differences in veterinarian and farm practices may have the potential to affect both endometrial swab findings and fertility outcomes in this population. However, the inclusion of farm as a random effect in all analyses will have accounted for some of this unobserved variation. Endometrial swab samples were taken in an unguarded method via a disposable vaginal speculum. While the use of a speculum will reduce the level of contamination from the vaginal vault, it is likely when compared, for example, to a double guarded swab, that the level of contamination may be higher with this method.^37^ Contamination of the vaginal vault is typically thought to produce a mixed colony of bacterial growth^38^ and since only monocultures were evaluated, present findings, particularly those with a profuse growth, are perhaps more likely to be considered by practitioners as being associated with acute endometritis. Additionally, the use of a publicly available data source to collect foaling outcomes could underestimate negative outcomes, which may be underreported at this level and, as with any study, missing data has the potential to introduce bias.

In conclusion, the results of this study highlight important, novel complexities around the interpretation of endometrial swab findings in Thoroughbred broodmares and demonstrate the importance of multivariable analyses for evaluation of fertility data, in particular the need to include mares' age and status, alongside potential measures of reproductive health. In young mares, endometrial inflammation (% PMN) appears less likely to impact fertility outcomes and, therefore, beyond the ruling out of venereal transmissible infections, an endometrial culture and cytology may be of somewhat limited value in young, clinically normal individuals. Conversely, the value of identifying and instituting effective reproductive management around the time of breeding in mares >12 years of age with evidence of a marked endometrial inflammatory response and mares with profuse *E. coli* cultures cannot be understated. Critically, and in contrast to previous studies,^11^, ^12^ through the evaluation of endometrial bacteriology species and growth separately, our findings have refined current knowledge and identified an important subset of mares with a profuse growth of *E. coli*, in which important knowledge gaps exist around the aetiologies underlying their poorer fertility outcomes. Further work to fill these gaps is urgently required, in order that appropriate and targeted treatment can be instigated and antimicrobial stewardship adhered to.

## FUNDING INFORMATION

This research received no external funding.

## CONFLICT OF INTEREST STATEMENT

The authors declare no conflicts of interest.

## AUTHOR CONTRIBUTIONS

**Billy Fehin:** Data curation; writing – original draft; conceptualization; investigation; methodology; writing – review and editing; validation; visualization. **Camilla J. Scott:** Data curation; project administration; writing – review and editing; conceptualization; investigation; writing – original draft; methodology; validation; visualization. **Juan Carlos Arango‐Sabogal:** Visualization; validation; writing – review and editing; methodology. **Amanda M. de Mestre:** Project administration; resources; supervision; writing – review and editing; conceptualization; validation; visualization; methodology. **Rebecca Mouncey:** Writing – original draft; formal analysis; methodology; validation; visualization; writing – review and editing; supervision; resources; project administration; software; conceptualization; investigation.

## DATA INTEGRITY STATEMENT

Rebecca Mouncey confirms that she had full access to all the data in the study and takes full responsibility for the integrity of the data and the accuracy of the data analysis.

## ETHICAL ANIMAL RESEARCH

Ethical approval was granted by the Royal Veterinary College's Clinical Research Ethical Review Board (URN: SR2019‐0472).

## INFORMED CONSENT

Explicit owner consent for inclusion of animals in this study was not obtained. Owners or their agents were made aware that case information may be used for research in general.

## Supporting information


**Figure S1.** Flow chart describing data availability and data exclusions from the initial database to the study sample and final multivariable model.

**Figure S2.** Plots demonstrating homoscedasticity and normality of the residuals (at the highest level; farm) of the final multivariable model.

**Figure S3.** Plot of the sum of the predicted against observed values from the final model for each decile of predicted probability.

**Table S1.** Distribution by year of 7691 last endometrial swabs taken from 3579 Thoroughbred mares on 196 stud farms during the northern hemisphere breeding season (15 February and 15 July) submitted to a laboratory in Newmarket, UK, between 2014 and 2020.

**Table S2.** The distribution of endometrial swab bacteriology and cytology findings for the most frequently reported categories of bacterial isolate from 7691 last endometrial swabs taken from 3579 Thoroughbred mares on 196 stud farms during the northern hemisphere breeding season (15 February and 15 July) submitted to a laboratory in Newmarket, UK, between 2014 and 2020.

**Table S3.** Distribution of reported outcomes by year for 3579 Thoroughbred mares on 196 stud farms that submitted 7691 endometrial swab samples during the northern hemisphere breeding season (15 February and 15 July) to a laboratory in Newmarket, UK, between 2014 and 2020.

**Table S4.** Results of mixed effects univariable regression analyses to investigate associations between endometrial swab findings and live foal rates in 3579 Thoroughbred mares on 196 stud farms that submitted 7691 endometrial swab samples during the northern hemisphere breeding season (15 February and 15 July) to a laboratory in Newmarket, UK, between 2014 and 2020.

## Data Availability

The data that support the findings of this study are available upon reasonable request from the corresponding author. Open data sharing exemption granted by the editor.
