# Supplementary material for: Associations between endometrial swab bacteriology and cytology findings and live foal rates in Thoroughbred broodmares in the United Kingdom
Source: Equine Vet J. 2025 Sep 1;58(2):348–58. doi: 10.1111/evj.70086 (PMC12892376; doi:10.1111/evj.70086)

Equine Veterinary Journal BEVA

Figure S1: Flow chart describing data availability and data exclusions from the initial database to the study sample and final multivariable model.

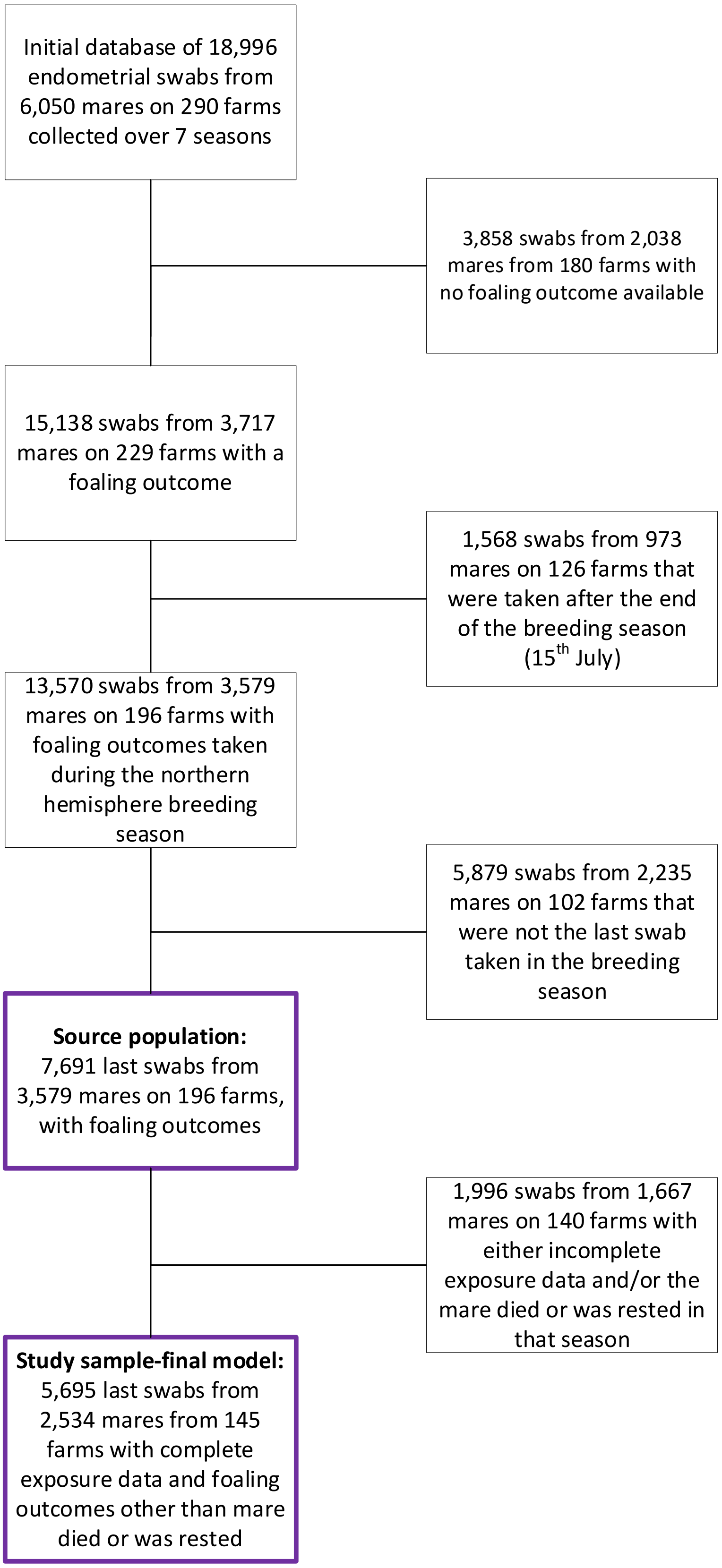

Supplement: Supplementary file 1 — Figure S1. Flow chart describing data availability and data exclusions from the initial database to the study sample and final multivariable model. [file EVJ-58-348-s005.pdf]
