# Supplementary material for: Associations between endometrial swab bacteriology and cytology findings and live foal rates in Thoroughbred broodmares in the United Kingdom
Source: Equine Vet J. 2025 Sep 1;58(2):348–58. doi: 10.1111/evj.70086 (PMC12892376; doi:10.1111/evj.70086)

Equine Veterinary Journal

BEVA

Figure S2: Plots demonstrating homoscedasticity and normality of the residuals (at the highest level; farm) of the final multivariable model.

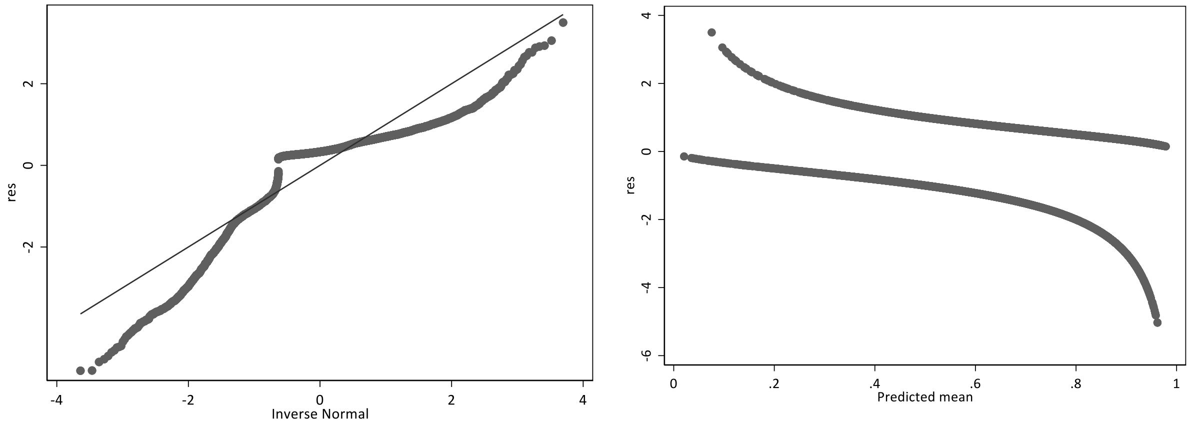

Supplement: Supplementary file 2 — Figure S2. Plots demonstrating homoscedasticity and normality of the residuals (at the highest level; farm) of the final multivariable model. [file EVJ-58-348-s004.pdf]
