# Supplementary material for: Associations between endometrial swab bacteriology and cytology findings and live foal rates in Thoroughbred broodmares in the United Kingdom
Source: Equine Vet J. 2025 Sep 1;58(2):348–58. doi: 10.1111/evj.70086 (PMC12892376; doi:10.1111/evj.70086)

Equine Veterinary Journal
BEVA

Figure S3: Plot of the sum of the predicted against observed values from the final model for each decile of predicted probability.

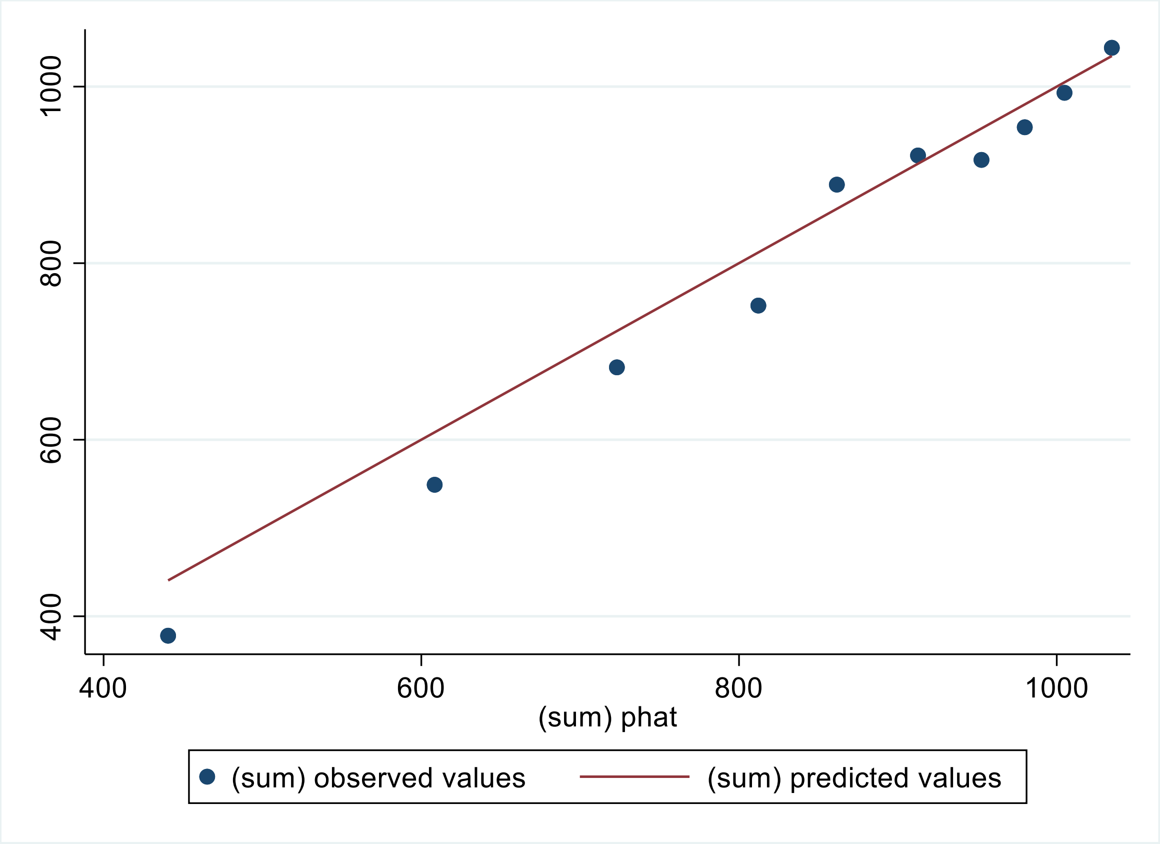

Supplement: Supplementary file 3 — Figure S3. Plot of the sum of the predicted against observed values from the final model for each decile of predicted probability. [file EVJ-58-348-s002.pdf]
