# Supplementary material for: Associations between endometrial swab bacteriology and cytology findings and live foal rates in Thoroughbred broodmares in the United Kingdom
Source: Equine Vet J. 2025 Sep 1;58(2):348–58. doi: 10.1111/evj.70086 (PMC12892376; doi:10.1111/evj.70086)
Supplement: Supplementary file 4 — Table S1. Distribution by year of 7691 last endometrial swabs taken from 3579 Thoroughbred mares on 196 stud farms during the northern hemisphere breeding season (15 February and 15 July) submitted to a laboratory in Newmarket, UK, between 2014 and 2020. [file EVJ-58-348-s003.pdf]

Table S1: Distribution by year of 7,691 last endometrial swabs taken from 3,579 Thoroughbred mares on 196 stud farms during the northern hemisphere breeding season (15 February and 15 July) submitted to a laboratory in Newmarket, UK, between 2014 and 2020.

Year	Swabs	Mares	Farms
2014	1,164	1,164	64
2015	1,098	1,098	57
2016	1,115	1,115	69
2017	1,116	1,116	74
2018	1,135	1,135	82
2019	1,176	1,176	75
2020	887	887	67
**All**	**7,691**	**3,579**	**196**
