# Supplementary material for: Associations between endometrial swab bacteriology and cytology findings and live foal rates in Thoroughbred broodmares in the United Kingdom
Source: Equine Vet J. 2025 Sep 1;58(2):348–58. doi: 10.1111/evj.70086 (PMC12892376; doi:10.1111/evj.70086)
Supplement: Supplementary file 5 — Table S2. The distribution of endometrial swab bacteriology and cytology findings for the most frequently reported categories of bacterial isolate from 7691 last endometrial swabs taken from 3579 Thoroughbred mares on 196 stud farms during the northern hemisphere breeding season (15 February and 15 July) submitted to a laboratory in Newmarket, UK, between 2014 and 2020. [file EVJ-58-348-s006.pdf]

Table S2: The distribution of endometrial swab bacteriology and cytology findings for the most frequently reported categories of bacterial isolate from 7,691 last endometrial swabs taken from 3,579 Thoroughbred mares on 196 stud farms during the northern hemisphere breeding season (15 February and 15 July) submitted to a laboratory in Newmarket, UK, between 2014 and 2020.

Bacteriology	0	+/-	1+	2+	3+	Total
**No Growth**	5,150	503	130	34	21	**5,838**
**BHS mono, few**	505	64	21	21	15	**626**
**BHS mono, moderate**	63	21	10	11	8	**113**
**BHS mono, profuse**	34	15	6	15	21	**91**
**EC mono, few**	265	24	8	1	4	**302**
**EC mono, moderate**	48	5	8	3	1	**65**
**EC mono, profuse**	42	8	2	0	3	**55**
**Total**	**6,107**	**640**	**185**	**85**	**73**	**7,090**

0(No PMN); +/− (≤0.5% PMN); 1+ (>0.5–5%PMN); 2+ (>5–30% PMN); 3+ (>30% PMN).
