# Supplementary material for: Associations between endometrial swab bacteriology and cytology findings and live foal rates in Thoroughbred broodmares in the United Kingdom
Source: Equine Vet J. 2025 Sep 1;58(2):348–58. doi: 10.1111/evj.70086 (PMC12892376; doi:10.1111/evj.70086)
Supplement: Supplementary file 6 — Table S3. Distribution of reported outcomes by year for 3579 Thoroughbred mares on 196 stud farms that submitted 7691 endometrial swab samples during the northern hemisphere breeding season (15 February and 15 July) to a laboratory in Newmarket, UK, between 2014 and 2020. [file EVJ-58-348-s007.pdf]

Equine Veterinary Journal BEVA

Table S3: Distribution of reported outcomes by year for 3,579 Thoroughbred mares on 196 stud farms that submitted 7,691 endometrial swab samples during the northern hemisphere breeding season (15 February and 15 July) to a laboratory in Newmarket, UK, between 2014 and 2020.

	Year							
**Outcome**	**2014**	**2015**	**2016**	**2017**	**2018**	**2019**	**2020**	**Total**
**Live foal**	919	889	944	935	994	996	680	6,048
**Barren**	179	142	158	154	144	145	122	916
**Aborted**	57	72	82	58	81	75	68	431
**Mare died**	27	15	20	19	16	14	17	117
**Dead foal**	8	16	15	8	19	8	3	75
**Rested**	7	31	18	23	26	14	16	104
**Total**	1,197	1,165	1,237	1,197	1,280	1,252	906	7,691

Barren; covered but not pregnant, aborted; pregnancy loss at any stage of gestation, rested; not covered.
