# Supplementary material for: Associations between endometrial swab bacteriology and cytology findings and live foal rates in Thoroughbred broodmares in the United Kingdom
Source: Equine Vet J. 2025 Sep 1;58(2):348–58. doi: 10.1111/evj.70086 (PMC12892376; doi:10.1111/evj.70086)
Supplement: Supplementary file 7 — Table S4. Results of mixed effects univariable regression analyses to investigate associations between endometrial swab findings and live foal rates in 3579 Thoroughbred mares on 196 stud farms that submitted 7691 endometrial swab samples during the northern hemisphere breeding season (15 February and 15 July) to a laboratory in Newmarket, UK, between 2014 and 2020. [file EVJ-58-348-s001.pdf]

Equine Veterinary Journal BEVA

Table S4: Results of mixed effects univariable regression analyses to investigate associations between endometrial swab findings and live foal rates in 3,579 Thoroughbred mares on 196 stud farms that submitted 7,691 endometrial swab samples during the northern hemisphere breeding season (15 February and 15 July) to a laboratory in Newmarket, UK, between 2014 and 2020.

**Predictor**	**Category**	**OR**	**95% CI**	**Wald *P***	***P***
Number of previous endometrial swabs	0	*Ref*	-	-	<0.0001
	1	0.78	0.67-0.91	0.002	
	2	0.37	0.30-0.44	<0.001	
	3-10	0.15	0.12-0.19	<0.001	
Endometrial swab cytology	0	*Ref*	-	-	<0.0001
	+/-	0.83	0.68-1.02	0.07	
	1+	0.56	0.40-0.78	0.001	
	2+	0.49	0.30-0.80	0.005	
	3+	0.37	0.22-0.60	<0.001	
Status	Foaling	*Ref*	-	-	<0.0001
	Barren	1.18	0.96-1.43	0.10	
	Rested	2.08	1.65-2.60	<0.001	
	Maiden	1.55	0.86-2.77	0.14	
	Aborted	1.72	0.18-16.86	0.64	
Age[a]*		0.90	0.89-0.91	<0.001	<0.0001
Endometrial swab bacteriology	No growth	*Ref*	-	-	<0.0001
	BHS mono few	0.91	0.73-1.14	0.41	
	BHS mono mod	0.78	0.47-1.29	0.34	
	BHS mono prof	0.61	0.37-1.01	0.06	
	EC mono few	0.94	0.68-1.29	0.69	
	EC mono mod	0.37	0.21-0.64	<0.001	
	EC mono prof	0.29	0.16-0.51	<0.001	

[a]=continuous variable, OR=odds ratio, CI=confidence interval, *Ref*=reference category, BHS=Beta haemolytic *Streptococcus,* EC= *Escherichia coli*, mono=monoculture, few=few colonies, mod=moderate growth, prof=profuse growth.
